# Bilayer coatings for extension of the shelf life of fish fillets: Incorporating seaweed sulfated polysaccharides in chitosan‐alginate LbL structures

**DOI:** 10.1002/fsn3.3934

**Published:** 2024-01-03

**Authors:** Fatemeh Khorami, Sedigheh Babaei, Shahriyar Valizadeh, Mahmood Naseri, Mohammad‐Taghi Golmakani

**Affiliations:** ^1^ Department of Natural Resources and Environmental Engineering, School of Agriculture Shiraz University Shiraz Iran; ^2^ Food and Nutritional Sciences Program North Carolina Agricultural and Technical State University Greensboro North Carolina USA; ^3^ Department of Food Science and Technology, School of Agriculture Shiraz University Shiraz Iran

**Keywords:** chitosan, fucoidan, layer‐by‐layer coating, rainbow trout, *Sargassum angustifolium*

## Abstract

The aim of this study was to develop a new active coating of layer‐by‐layer (LbL) structure composed of alginate (as polyanions) and chitosan (as a polycation) containing sulfated polysaccharide (fucoidan) from *Sargassum angustifolium*, to protect rainbow trout fillets during refrigerated storage. Chitosan and alginate do not combine with each other as a homogeneous solution, so they are suitable for multilayer coatings. The results demonstrated that coating samples with chitosan and fucoidan significantly improved the quality of fish fillets and extended their shelf life from 6 to 16 days. The chemical values (TBARS and TVB‐N) and bacterial growth (total viable count (TVC), total psychrophilic count (PTC), and lactic acid bacteria (LAB)) indicated lower levels in the LbL coating samples containing fucoidan compared to the alginate and control samples. Among the different coating samples, the LbL coating with fucoidan (AChF1) exhibited lower weight loss, improved chromaticity (L*, a*, and b*), and minimal changes in mechanical and sensory evaluations. Based on the findings, AChF1 was the most effective treatment for increasing the shelf life of rainbow trout fillets during refrigerated storage. Therefore, it has potential applications in the food packaging industry.

## Graphical abstract

Marine sulfated polysaccharide from the *Sargassum angustifolium* from Persian Gulf has antioxidant and antibacterial potential in the seafood packaging industry. Multilayer packaging systems using a layer‐by‐layer (LbL) electrostatic deposition technique based on alginate (as polyanions) and chitosan (as a polycation) can increase the shelf‐life extension of fish fillets during refrigerated storage.

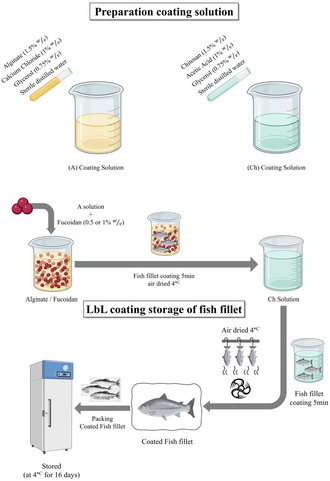

## 1 INTRODUCTION

Seafood plays a significant role in people's diets, offering high nutritional value with easily digestible protein, calories, and beneficial omega‐3 fatty acids (Huyben et al., 2018). However, the perishability of fish poses a challenge because of the existence of unsaturated fatty acids and enzymes that can lead to oxidation, resulting in changes in taste and quality (Gómez‐Estaca et al., 2009). Oxidation leads to the production of volatile compounds such as hydroperoxides, fatty acids, ketones, etc. which change the organoleptic properties (Feng et al., 2021; Fraga‐Corral et al., 2022). Packaging via edible coatings and biodegradable polymers containing antioxidant and antimicrobial compounds can protect aquatic products against water vapor, gases, mechanical and chemical damage, and microorganism activities (Moeini et al., 2022). Chitosan, a natural antimicrobial biopolymer, is obtained from the alkaline deacetylation of chitin (Wen et al., 2023). In fact, their active cationic groups (amine groups) react with the membrane of the cell of microorganisms and lead to the leakage and exiting of intracellular protein substances and finally the death of the microorganism (Valizadeh et al., 2019).

However, using single‐coating materials may not meet practical requirements, making the combination of chitosan with other polysaccharides a viable approach to enhancing their functional and physical properties (Kim et al., 2018). Alginate is another abundant marine polysaccharide that is found in brown seaweed. It has gel‐forming and antimicrobial properties (Abdallah et al., 2018), making it suitable for use as an external layer in food coatings.

The importance of using these products for food packaging has been doubled by enriching edible films and coatings with antimicrobial and antioxidant agents (Goulas et al., 2005). Sulfated polysaccharides like fucoidan from the cell wall of brown algae, carrageenan, and olvan from red and green algae, respectively (Wijesekara et al., 2011) have antioxidant (De Jesus Raposo et al., 2013; Vardizadeh et al., 2021) and antibacterial (Hayashi et al., 2008) properties because of the existence of sulfate agents as active groups in their structures. Fucoidan is a non‐toxic compound with a safe and secure structure (Elizondo‐Gonzalez et al., 2012). Roohinejad et al. (2017) found that the addition of seaweeds, in the form of raw material, their extracts, or isolated compounds, to food products can improve their shelf‐life. Additionally, sulfated polysaccharides obtained from Sargassum and Padina have demonstrated similar preservation effects to the commercial preservative BHT when storing fish oil under accelerated oxidation conditions. While there have been reports of fucoidan from seaweed being used in antioxidant edible films in various studies (Asad Samani et al., 2023), there is limited research on extending the shelf‐life of fish fillets using marine sulfated polysaccharides.

Multilayer packaging systems have emerged by using a layer‐by‐layer (LbL) electrostatic deposition method as a promising approach. This technique is based on the substitute deposition of oppositely charged electrolytes and offers improved coating properties such as reducing texture degradation, increased mechanical strength, shelf‐life extension, and higher barriers for both water and gases (Dong et al., 2018; Poverenov et al., 2014). Chitosan (as a polycation) and alginate and fucoidan (as polyanions) do not combine with each other and do not form a homogeneous solution (Kim et al., 2018), so they are suitable for multicomponent coatings. The aim of this study is to develop active bilayer films using alginate, chitosan, and sulfated polysaccharide from *Sargassum angustifolium* via the LbL technique to protect rainbow trout fillets from oxidation during refrigeration storage. One of the most important aims of this research is to investigate the chemical, microbial, mechanical, and sensory changes of rainbow trout fillets and also compare them with the control sample.

## 2 MATERIALS AND METHODS

### 2.1 Materials

Chitosan (MW 450 kDa, 90%–95% deacetylated), ethanol (96%), acetone, glycerol, PCA and MRS agar, magnesium hydroxide, boric acid (3%), sodium alginate, sulfuric acid (0.05 M), methylene blue, methyl red, hydrochloric acid, thiobarbituric acid, calcium chloride, and trichloroacetic acid were obtained from Sigma Aldrich (St. Louis, Missouri, USA), Merck Co. (Darmstadt, Germany), and Zakaria Tajhiz Parseh (Iran).

### 2.2 Algae preparation

*S. angustifolium*, a brown macroalga, was taken from Fars Science and Technology Park, Abdf Company‐Algae Bank (from the Persian Gulf coast), and after cleaning, it was washed several times with fresh water. The macroalgae was dried using an oven at 40°C. Afterward, they were ground into a powder using a coffee grinder, passed through a No. 50 sieve, and stored at −20°C (Ibrahim & Lim, 2015).

### 2.3 Extraction of sulfated polysaccharide

High antioxidant properties from the sulfated polysaccharide (SP) of *Sargassum* were previously reported by Vardizadeh et al. (2021), which inhibit fish oil oxidation during accelerated oxidation conditions. So, SP was extracted based on the Vardizadeh et al. (2021) method. Briefly, pigments and fat were removed using ethanol, the supernatant was removed by using a Büchner funnel, and the precipitated alga was washed several times using acetone. The decolorized algae were mixed with distilled water and heated at 65–70°C for 3 h. After this step, it was centrifuged for 10 min at 5000 rpm at room temperature. Next, the supernatant was collected and concentrated. Calcium chloride was used to remove alginate. Afterward, cold ethanol (96%) was added to the obtained supernatant and maintained in the refrigerator overnight. The precipitated SPs were separated using a centrifuge (5000 rpm, 10 min, and 25°C) and washed with acetone and ethanol (96%). Eventually, the precipitated SPs were dried.

### 2.4 Fish fillet preparation

The rainbow trout (*Oncorhynchus mykiss*) (30 fish around 400 g in weight) were obtained from a fish farm. The fish were moved to a Packaging and Processing Factory (Liossa) in special boxes with ice, and then they were washed and filleted. After being filleted, they were covered with ice and transferred to the seafood laboratory.

### 2.5 Treatments and preparation of LbL coating

Five treatments were prepared in this study, including (C) negative control, (A) alginate coating, (Ch) chitosan coating, (ACh) alginate‐chitosan LbL coating, (AChF0.5) alginate/fucoidan (0.5%, W:V)‐chitosan LbL coating, and (AChF1) alginate/fucoidan (1%, W:V)‐chitosan LbL coating.

For preparation of the alginate coating solution (A), sodium alginate powder (1.5%, W:V) was dissolved in sterile distilled water and hydrated at 50°C for 4 h, and calcium chloride (1%, W:V) was added for gelation. Chitosan coating solution (Ch) (1.5%, W:V and acetic acid (1%, W:V)) was dissolved in 100 mL of sterile distilled water and stirred on a stirrer hot plate at 25°C for 4 h. Glycerol (0.75%, W:V) was added to each of the solutions, separately, as a plasticizer. After the solutions were cooled on the side of the ice, fish fillets (40–50 g) were dipped in the solutions for 5 min. Then samples were put in an incubator for 10 min at 4°C to remove the excess solution from the fillets.

For preparation of the alginate‐chitosan LbL coating (ACh), fillets coated with alginate (A) were dipped in the solution of chitosan for 5 min and air‐dried in the incubator, as mentioned above (Kim et al., 2018). To prepare the AChF0.5 and AChF1 LbL coatings, first, fucoidan was added to the alginate solution and thoroughly stirred to obtain a final concentration of 0.5% and 1% (W:V). Then, fillets were submerged in an alginate/fucoidan solution for 5 minutes and air‐dried in the incubator for 10 min at 4°C. Then they were immersed in chitosan solution (Ch) using the same method. All samples were put in the incubator at 4°C to remove the excess solution from the fillets and create the desired coating on their surface. Fillets that are not coated were used as the negative control (C). All samples were packaged in sterile polyethylene bags, placed on trays, and stored at 4 ± 1°C for 16 days. Sampling was done randomly on 0, 4, 8, 12, and 16 days. On each sampling day, 6 fillets were separated from each treatment to analyze quality indices (Yu et al., 2018).

### 2.6 Chemical analysis

#### 2.6.1 pH value

To measure the pH of the samples, 5 g of minced fillet was homogenized with 50 mL of distilled water for 30 min. Then, the pH value of samples was detected using a pH meter (Ohaus Co., Switzerland) (Li et al., 2017).

#### 2.6.2 Total volatile base nitrogen (TVB‐N)

The TVB‐N of samples was measured using the Cai et al. (2014) method with a few modifications. In brief, a 10‐g homogenized sample was distilled in a Kjeldahl balloon comprising 50 mL of distilled water and 2 g of magnesium oxide. The mixture was boiled for 20 min, and the resulting distilled extract was collected in boric acid (3%) containing methyl‐red and methylene blue as the indicators, and finally, it was titrated with sulfuric acid (0.05 M). The total volatile basic nitrogen (TVBN) was determined three times, and the results were expressed as mg N/100 g fish fillet.

#### 2.6.3 Thiobarbituric acid reactive substance determination (TBARS)

The thiobarbituric acid reactive substances (TBARS) value in the samples was determined as per the method (Nirmal & Benjakul, 2010). Briefly, 1 g of minced fish fillet was mixed with 9 mL of hydrochloric acid (0.25%), which contained thiobarbituric acid (0.375%; w/v) and trichloroacetic acid (15%; w/v). This mixture was put in a bain‐marie bath at 80°C for 10 min. Then, after cooling with cold water, it was centrifuged at 4000 rpm for 20 minutes. The absorbance of the supernatant of the samples was read at 532 nm using a spectrophotometer (T70 UV/VIS Spectrometer, England).

### 2.7 Physical properties

#### 2.7.1 Weight loss

According to the Vital et al. (2018) method, the weight loss (WL) of fish fillet samples was measured. The weight of each sample was recorded on each evaluation day, and WL was measured based on the following formula:

(1) $$\mathrm{WL}\;\left(\%\right)=\frac{\left(\mathrm{wi}-\mathrm{wf}\right)}{\mathrm{wi}}\times 100$$

where *wi* = initial weight and *wf* = final weight (on 4, 8, 12, and 16 days).

#### 2.7.2 Texture analysis

The fillet texture was measured according to a method explained by Bourne (1978) with some modifications, using a Texture Analyzer (TAXT‐2i, Stable Microsystems, Surrey, England). The speed was 1 mm/s, the distance between cylindrical rods was 20 mm, and the cylindrical rod was 10 g. The 2.0 × 2.0 cm^2^ fillets were prepared and cut from the anterior region to the dorsal fin which is above the lateral line of the fillets.

#### 2.7.3 Chromaticity parameters

Color features of films were investigated in a smart colorimeter (MAT, 2000 Series; IDME Co., Ltd., Shiraz, Iran) based on scales of CIELab. To obtain this aim, the coated fillet samples were put inside the color meter, and three factors, L* (lightness), a* (red‐green), and b* (yellow‐blue), were determined based on the Lu et al. (2010) method. Since the color of the fish fillet is not uniform on the whole surface, three specific points were measured: anterior, middle, and posterior. Control and treatments were examined six times, and their average was used.

### 2.8 Microbial analysis

The bacterial count of the samples was investigated using the method explained by Raeisi et al. (2016). Briefly explained, 10 g of fish fillet samples were homogenized with 90 mL of sterile saline solution (0.85%) using a sterile instrument for 3 minutes. Different dilutions of the homogenized samples were prepared by serial dilution. Then, they were transferred to plate count and MRS agar by the pour plate method. Finally, they were placed in an incubator at 37°C (total viable count (TVC)), 25°C (lactic acid bacteria (LAB)), and 7°C (total psychrophilic count (TPC)) for 3, 5, and 7 days, respectively.

### 2.9 Sensory evaluation

Semi‐trained people did the sensory analysis for the fish fillets according to 5 hedonic point scales. The texture, color, odor, and complete acceptability of the fillets were analyzed by the panelists. Level 1 was considered for the lowest quality and Level 5 for the highest quality (Joukar et al., 2017).

### 2.10 Statistical analysis

The statistical analysis of the data was carried out using SPSS software (Ver. 25.0, IBM Corp., Armonk, NY, USA). At least three replications were carried out for each test. First, the normality of the data and the homogeneity of the variance were checked by Levene's and Shapiro–Wilk's tests, respectively. Thereafter, a one‐way ANOVA was used to evaluate the significant differences between the samples. Duncan's multiple range test was performed to detect the significant differences in the means at a confidence level of 95%. Cross‐Calvalis and Mann–Whitney U tests were done for non‐parametric results (sensory analysis). Excel (version 2019) software was used to draw graphs. The results are reported as mean ± *SD*.

## 3 RESULTS AND DISCUSSION

### 3.1 Chemical analysis

#### 3.1.1 pH value

**FIGURE 1:**
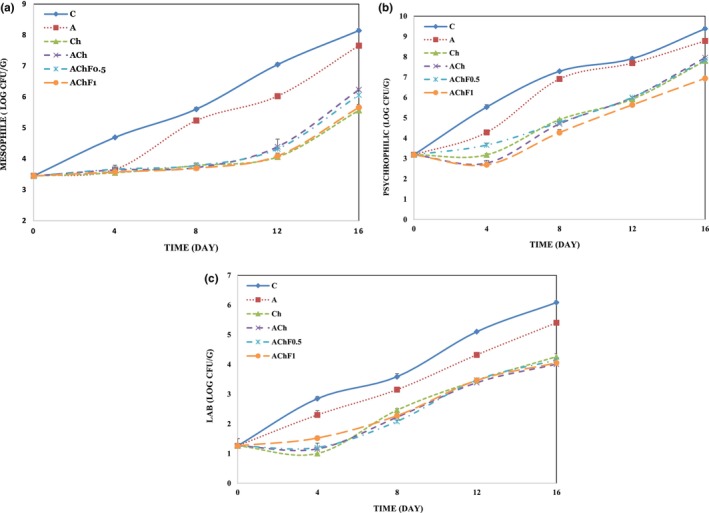
Changes in total viable counts (TVC) (a), total psychrophilic count (TPC) (b), and lactic acid bacteria (LAB) (c) of fish samples during refrigerated storage. C: uncoated fish fillet (negative control), A: fish fillet coated with alginate, Ch: fish fillet coated with chitosan, ACh: fish fillet coated with alginate‐chitosan LbL coating, AChF0.5: fish fillet coated with alginate/fucoidan (0.5%)‐chitosan LbL coating, AChF1: fish fillet coated with alginate/fucoidan (1%)‐chitosan LbL coating. (*n* = 3, Mean ± *SD*).

The pH values of fish fillets along the experiment period are shown in Table 1. Initially, the minced fish fillet had a pH value of 6.6. Typically, the pH of marine organisms decreases after death because of lactic acid production along the glycolysis process, usually ranging from 5.5 to 6.5 (Lougovois & Kyrana, 2005). On the fourth day, the pH values of the different treatments initially decreased, which was attributed to the CO_2_ dissolving in the minced fish fillets and the acid produced during the glycogen decomposition process in the samples. There was not any statistically significant difference (*p* < .05) among the treatments on the fourth day, likely because of the production of volatile bases and internal enzymatic or microbial reactions (Chamanara et al., 2013). Throughout the entire period, the pH values of the AChF0.5 and AChF1 treatments were significantly lower than the other treatments (*p* < .05). This can be attributed to the antimicrobial properties of fucoidan (Figure 1) and its ability to inhibit enzyme activity (Arfat et al., 2015). Similar results were observed in chicken fillet coated with chitosan and pulled‐out propolis (Jafari et al., 2018) and rainbow trout coated with chitosan and mixed with pomegranate peel extract (Berizi et al., 2018).

**TABLE 1:** Chemical quality indices of fish fillet during refrigerated storage.

Test	Treatment	Storage time (day)				
		0	4	8	12	16
pH	C	6.60 ± 0.01^Ac^	6.43 ± 0.07^ABa^	6.58 ± 0.02^Ac^	6.78 ± 0.06^Ab^	6.90 ± 0.01^Aa^
	A	6.60 ± 0.01^Ac^	6.48 ± 0.01^Ad^	6.61 ± 0.01^Ac^	6.74 ± 0.03^Ab^	6.83 ± 0.03^Ba^
	Ch	6.60 ± 0.01^Aa^	6.37 ± 0.01^Bc^	6.50 ± 0.08^Bb^	6.52 ± 0.04^BCb^	6.67 ± 0.02^Da^
	ACh	6.60 ± 0.01^Ab^	6.44 ± 0.01^Ad^	6.46 ± 0.01^Bd^	6.51 ± 0.01^BCc^	6.72 ± 0.04^Ca^
	AChF0.5	6.60 ± 0.01^Ac^	6.37 ± 0.01^Bd^	6.46 ± 0.02^BCc^	6.47 ± 0.01^Cc^	6.66 ± 0.01^DEa^
	AChF1	6.60 ± 0.01^Aa^	6.42 ± 0.03^ABc^	6.39 ± 0.03^Bc^	6.55 ± 0.03^Bb^	6.62 ± 0.03^Ea^
TBARS (mg MDA/kg lipid)	C	0.78 ± 0.10^Ae^	2.13 ± 0.06^Ad^	13.96 ± 0.39^Ac^	16.09 ± 1.07^Ab^	23.99 ± 0.14^Aa^
	A	0.78 ± 0.10^Ae^	1.88 ± 0.05^Bd^	11.99 ± 0.62^Bc^	14.98 ± 0.77^Ab^	22.78 ± 0.62^Ba^
	Ch	0.78 ± 0.10^Ad^	1.92 ± 0.04^Bc^	9.73 ± 0.43^Cb^	10.14 ± 0.53^Bb^	11.94 ± 0.66^Ca^
	ACh	0.78 ± 0.10^Ae^	1.80 ± 0.08^Bd^	9.02 ± 0.37^Cc^	9.98 ± 0.54^Bb^	10.99 ± 0.25^Da^
	AChF0.5	0.78 ± 0.10^Ad^	1.55 ± 0.09^Cc^	6.78 ± 0.25^Db^	7.90 ± 0.40^Ca^	8.03 ± 0.48^Ea^
	AChF1	0.78 ± 0.10^Ad^	1.33 ± 0.05^Dc^	6.87 ± 0.24^Db^	8.57 ± 0.12^Ca^	8.58 ± 0.11^Ea^
TVB‐N (mg N/100 g fish)	C	6.30 ± 0.70^Ae^	12.04 ± 0.14^Ad^	15.26 ± 0.52^Ac^	20.17 ± 0.91^Ab^	32.20 ± 1.40^Aa^
	A	6.30 ± 0.70^Ae^	11.90 ± 0.00^Ad^	15.05 ± 0.58^Ac^	19.31 ± 0.87^Ab^	30.12 ± 0.65^Ba^
	Ch	6.30 ± 0.70^Ad^	10.78 ± 0.28^Cc^	13.02 ± 0.14^Bb^	14.00 ± 0.00^Ca^	14.42 ± 0.14^Da^
	ACh	6.30 ± 0.70^Ad^	11.24 ± 0.09^Bc^	13.30 ± 0.00^Bb^	16.10 ± 0.42^Ba^	16.45 ± 0.35^Ca^
	AChF0.5	6.30 ± 0.70^Ad^	9.80 ± 0.14^Ec^	12.81 ± 0.07^Bb^	13.93 ± 0.07^Ca^	14.28 ± 0.00^Da^
	AChF1	6.30 ± 0.70^Ae^	10.43 ± 0.07^Dd^	11.83 ± 0.21^Cc^	12.67 ± 0.07^Dd^	13.44 ± 0.14^Da^

*Note*: The effect of storage time on each treatment is shown in lowercase letters. The comparison of different treatments is shown in capital letters (*p* < .05). C: uncoated fish fillet (negative control), A: fish fillet coated with alginate, Ch: fish fillet coated with chitosan, ACh: fish fillet coated with alginate‐chitosan LbL coating, AChF0.5: fish fillet coated with alginate/fucoidan (0.5%)‐chitosan LbL coating, AChF1: fish fillet coated with alginate/fucoidan (1%)‐chitosan LbL coating (*n* = 3, Mean ± *SD*).

#### 3.1.2 TVB‐N

Besides taste, color, and appearance, the TVB‐N content serves as a crucial indicator of meat product freshness (Weng et al., 2020). When meat products undergo microbial spoilage, volatile base compounds such as ammonia, monoethylamine, diethylamine, and triethylamine are generated (Rodrigues et al., 2013). It has been announced that the maximum acceptable TVB‐N limitation in fish fillets is 30 mg N/100 g sample. Table 1 presents TVB‐N level changes for various samples during storage. Initially, the average TVB‐N amount on the first day was 6.3 mg N/100 g fish fillet, which increased over time. Generally, the C and A samples exhibited significantly higher values of TVB‐N compared to the other samples. Among the storage times, the AChF1 sample displayed the lowest TVB‐N value. The rise in TVB‐N can be attributed to the activity of spoilage bacteria and internal enzymes, resulting in the production of ammonia and amines (I, II, and III types) (Berizi et al., 2018). The microbial results (Figure 1) indicate a direct correlation between microbial growth and the TVB‐N value. The AChF1 sample, with the lowest microbial growth, also exhibited the lowest TVB‐N value. Bilgin and Gençcelep (2015) reported that the highest acceptable limit for TVB‐N in fish is 30 mg N/100 g fillet. After 16 days of storage, the control and A treatments reached critical levels of TVB‐N (32.20 and 30.12 mg N/100 g fish, respectively), while the AChF0.5 and AChF1 samples had the lowest amounts at 13.44 and 14.28 mg N/100 g fish, respectively. Xiong et al. (2021) discovered that incorporating chitosan and alginate with gallic acid as a coating significantly prevented salmon protein degradation. However, Feng et al. (2016) showed that integrating chitosan and gelatin enhanced the antimicrobial properties of the coating, decreased microbial counts, and hindered oxygen permeation, consequently lowering the TVB‐N value.

#### 3.1.3 TBARS

The TBARS index is an important measure of secondary lipid oxidation products, specifically malondialdehyde (MDA), and is commonly used to assess oxidative deterioration in food products. It is influenced by factors such as microbial spoilage and oxygen‐induced oxidation during storage (Rathod et al., 2021). The values of TBARS (mg MDA/kg sample) of samples along the storage period are illustrated in Table 1. The first TBARS value of the samples was 0.78 mg MDA/kg, and it went up gradually over time during storage. During storage period, the value of TBARS in the control and A samples was significantly higher than in other samples, which can be attributed to relative fish dehydration, fillet, and unsaturated fatty acid oxidation (Al‐Qurashi & Awad, 2018). The findings suggest that chitosan coatings effectively prevent the oxidation of lipid in fish fillets. Chitosan was found to inhibit the increase in the TBARS index in cod (*Gadus morhua*) fillets (Inanli et al., 2020). Ojagh et al. (2010) and Sallam (2007) reported that chitosan had antioxidant properties and could prevent oxygen penetration in fish fillets. According to the Mol et al.'s (2023) study, TBARS values of 3, 5, and 8 mg MDA/kg sample are considered excellent, good, and acceptable limits, respectively. From the 8th to the 16th day of storage, only the AChF0.5 and AChF1 samples maintained TBARS values within the acceptable range compared to the other treatments. The AChF0.5 and AChF1 samples showed the lowest TBARS throughout the storage period. The effectiveness of fucoidan in preventing lipid oxidation and its antioxidant properties have been demonstrated by Loayza‐Gutiérrez et al. (2022). Polysaccharides containing hydrogen donor groups, such as –COOH and –SO_3_H, have been shown to enhance the capacity to scavenge free radicals (Vardizadeh et al., 2021).

### 3.2 Physical properties

#### 3.2.1 Weight loss

Coatings are crucial in preventing weight loss in food products, as this helps to preserve water‐soluble nutrients, particularly proteins and amino acids, and has economic benefits (Shahidi & Hossain, 2022). Table 2 displays the weight loss percentage of coated fillets during storage at 4°C in a refrigerator. There were no important differences in weight loss among different samples up to day 8 (*p* < .05). Moreover, there was no significant difference between Ch, ACh, AChF0.5, and AChF1 samples at 12 and 16 days. Finally, after the storage period, the A and control samples had a weight loss of around 7%, while the AChF1 sample had a weight loss of 2.5%. The results indicated that coatings with chitosan had a better ability to retain tissue water. Vital et al. (2018) reported similar findings when *Oreochromis niloticus* fish fillets coated with alginate‐containing plant essential oils showed a higher percentage of weight loss. Feng et al. (2016) also found that fish fillets coated with gelatin had lower weight loss than those coated with chitosan. This may be due to the high ability of gelatin to maintain moisture and its insulation against water passage.

**TABLE 2:** Mechanical and color changes (*L*, *a* & *b*) of fish fillet during refrigerated storage.

Test	Treatment	Storage time (day)				
		0	4	8	12	16
Wight loss	C	0.00 ± 0.00^Ad^	0.47 ± 0.09^Acd^	1.04 ± 0.22^Ac^	2.97 ± 0.27^Ab^	7.34 ± 1.08^Aa^
	A	0.00 ± 0.00^Ae^	0.47 ± 0.10^Ad^	0.99 ± 0.14^Ac^	2.82 ± 0.45^Ab^	5.81 ± 0.20^Ba^
	Ch	0.00 ± 0.00^Ad^	0.40 ± 0.05^Acd^	0.86 ± 0.19^Abc^	1.29 ± 0.03^Bb^	3.29 ± 0.56^Ca^
	ACh	0.00 ± 0.00^Ad^	0.50 ± 0.01^Acd^	0.93 ± 0.20^Abc^	1.43 ± 0.32^Bb^	2.96 ± 0.83^Ca^
	AChF0.5	0.00 ± 0.00^Ad^	0.41 ± 0.12^Ac^	0.97 ± 0.01^Ab^	1.23 ± 0.12^Bb^	2.41 ± 0.29^Ca^
	AChF1	0.00 ± 0.00^Ac^	0.39 ± 0.09^Abc^	0.81 ± 0.12^Abc^	1.17 ± 0.15^Bb^	2.54 ± 1.21^Ca^
Firmness (N)	C	138.4 ± 61.2^Ab^	326.8 ± 5.3^Aa^	342.3 ± 12.6^Aa^	346.1 ± 7.9^Aa^	345.8 ± 9.1^Aa^
	A	138.4 ± 61.2^Ab^	271.5 ± 8.9^Ba^	289.9 ± 19.7^Ba^	281.4 ± 10.3^Ba^	322.0 ± 10.6^Ba^
	Ch	138.4 ± 61.2^Ac^	245.6 ± 10.5^Cb^	269.7 ± 15.9^BCab^	281.9 ± 11.2^Bab^	303.4 ± 8.5^Ca^
	ACh	138.4 ± 61.2^Ac^	257.0 ± 10.6^Cb^	253.5 ± 22.1^Cb^	290.7 ± 10.0^Bab^	317.0 ± 8.8^BCa^
	AChF0.5	138.4 ± 61.2^Ac^	194.1 ± 9.1^Db^	203.2 ± 12.2^Dab^	229.1 ± 13.5^Cab^	252.9 ± 8.1^Da^
	AChF1	138.4 ± 61.2^Ac^	180.3 ± 5.0^Dbc^	193.2 ± 9.4^Dab^	223.9 ± 11.3^Cab^	244.9 ± 10.2^Da^
*L*	C	53.79 ± 5.4^Aa^	53.95 ± 2.5^BCDa^	47.61 ± 2.7^Bb^	48.97 ± 3.3^Cb^	56.85 ± 1.8^BCa^
	A	53.79 ± 5.4^Ab^	57.04 ± 2.8^ABb^	55.66 ± 1.9^Ab^	57.29 ± 5.0^Ab^	62.85 ± 3.3^Aa^
	Ch	53.79 ± 5.4^Aab^	51.98 ± 2.0^CDab^	49.09 ± 2.2^Bb^	50.03 ± 7.3^BCab^	56.25 ± 5.9^BCa^
	ACh	53.79 ± 5.4^Ab^	58.08 ± 3.7^Aab^	56.94 ± 1.4^Aab^	55.51 ± 3.8^ABab^	59.30 ± 3.2^ABa^
	AChF0.5	53.79 ± 5.4^Aab^	55.52 ± 2.5^ABCa^	50.10 ± 2.1^Bb^	54.94 ± 1.1^ABCa^	52.70 ± 3.4^CDa^
	AChF1	53.79 ± 5.4^Aa^	50.98 ± 3.8^Da^	49.31 ± 2.6^Ba^	50.57 ± 5.4^BCa^	50.88 ± 5.2^Da^
*a*	C	4.05 ± 2.89^Aa^	3.03 ± 0.69^Aab^	2.50 ± 2.13^ABab^	2.15 ± 1.34^Aab^	1.57 ± 0.98^Ab^
	A	4.05 ± 2.89^Aa^	2.61 ± 1.77^Aab^	1.94 ± 1.90^ABab^	1.36 ± 0.94^Ab^	2.16 ± 1.07^Aab^
	Ch	4.05 ± 2.89^Aa^	3.44 ± 1.47^Aa^	3.06 ± 0.60^Aa^	2.20 ± 1.66^Aa^	2.11 ± 2.22^Aa^
	ACh	4.05 ± 2.89^Aa^	2.06 ± 1.52^Aab^	1.04 ± 0.86^Bb^	1.44 ± 1.56^Ab^	2.43 ± 0.84^Aab^
	AChF0.5	4.05 ± 2.89^Aa^	2.87 ± 0.87^Aab^	1.62 ± 0.99^ABb^	1.99 ± 0.58^Aab^	2.47 ± 1.81^Aab^
	AChF1	4.05 ± 2.89^Aa^	3.05 ± 0.89^Aa^	2.90 ± 1.07^Aa^	2.32 ± 2.43^Aa^	2.96 ± 2.01^Aa^
*b*	C	19.56 ± 1.84^Abc^	22.04 ± 1.05^Aa^	19.97 ± 0.61^BCb^	18.95 ± 0.96^Dbc^	18.11 ± 1.06^Cc^
	A	19.56 ± 1.84^Ab^	20.86 ± 0.59^ABCa^	21.54 ± 0.53^Aa^	21.16 ± 0.83^BCa^	19.12 ± 0.91^BCb^
	Ch	19.56 ± 1.84^Aa^	19.15 ± 1.53^Ca^	18.93 ± 1.01^CDa^	19.52 ± 2.46^ABa^	20.46 ± 3.00^ABa^
	ACh	19.56 ± 1.84^Ab^	21.51 ± 1.57^ABa^	21.76 ± 1.53^Aa^	22.33 ± 1.26^ABa^	22.05 ± 1.49^Aa^
	AChF0.5	19.56 ± 1.84^Ac^	22.47 ± 1.79^Aab^	22.31 ± 1.05^Aab^	23.52 ± 1.79^Aa^	21.1 ± 0.60^ABbc^
	AChF1	19.56 ± 1.84^Ab^	19.92 ± 2.12^BCb^	20.91 ± 1.76^ABab^	22.61 ± 1.92^ABa^	19.9 ± 2.4^ABCb^

*Note*: The effect of storage time on each treatment is shown in lowercase letters. The comparison of different treatments is shown in capital letters (*P* < .05). C: uncoated fish fillet (negative control), A: fish fillet coated with alginate, Ch: fish fillet coated with chitosan, ACh: fish fillet coated with alginate‐chitosan LbL coating, AChF0.5: fish fillet coated with alginate/fucoidan (0.5%)‐chitosan LbL coating, AChF1: fish fillet coated with alginate/fucoidan (1%)‐chitosan LbL coating (*n* = 3, Mean ± *SD*).

#### 3.2.2 Texture analysis

Fish texture is an important influencing factor in product quality and its marketability. Thus, the evaluation of fish texture is important for determining the effect of its storage method on product quality. Fish tissue quality depends on various internal factors like the collagen amount, myofibril, autolysis, and microbiological processes (Vilkova et al., 2022). Based on the Tokay et al. (2021) study, the first stage of loss of quality of fish texture is attributed to enzymatic autolysis and the second stage to microbial activity and microorganisms, which have an important impact on quality loss, color, and smell of marine products. As shown in Table 2, The texture of AChF1 and AChF0.5 samples had the lowest firmness during storage time. There is a direct relationship between weight loss and the firmness of the fillet textures, as shown in Table 2. It means that the coatings containing fucoidan could prevent the exit of water from the product tissue. Since the microbial index affects the fish texture quality (Zhuang et al., 2023), the quality of texture maintained in AChF0.5 and AChF1 samples can be due to the low rate of microorganisms' growth (Figure 1). The results were similar to Chamanara et al. (2012) report about the usage of chitosan and *Thymus vulgaris* essential oil coating on the shelf life of fish fillet. Moreover, Cao et al. (2020) reported that chitosan coatings containing chlorogenic acid increased the hardness and stiffness of snakehead fish fillets during the refrigerator storage period.

### 3.3 Chromaticity parameters

Chromaticity parameter results (L*, a*, and b*) of the samples along the storage period in the refrigerator are shown in Table 2. There was not any statistically important difference in L* (lightness), b* (yellow‐blue), and a* (red‐green) values between treatments during the storage period. But, a* and b* values were slightly decreased in the uncoated sample during and at the end of the period. Our results were similar to Hai et al.'s (2022) results on *Oreochromis niloticus* alginate‐coated fillets containing clove essential oil and Xiong et al.'s (2021) results on salmon fillets coated with salmon bone gelatin containing clove essential oil.

The a* (red‐green) and b* value (yellow‐blue) indices in meat products are common indices for evaluating their freshness. Red meats such as beef and pork have a higher a* index, but in fish, it is different from one species to another (Yu et al., 2018). The initial average a* value in the samples was 4.05, as shown in Table 2, which decreased within the storage period, especially for uncoated ones. The reason for the change in color of meat can be attributed to red oxymyoglobin oxidation pigment and converting them to brown metmyoglobin, which made fish fillets darker (Li et al., 2017). The lightness of fish fillets is affected by different parameters like pigments, pH, protein denaturation, lipid oxidation, the physical structure of the muscles, water content, and microbial spoilage (Cheret et al., 2005; Yu et al., 2018). Moreover, changes in the fish color along the storage period are related to reactions that are non‐enzymatic (microorganisms and oxidation) and enzymes that lead to the destruction of myofibril protein and the disintegration of myofibrils, which leads to changes in the meat appearance (Cheret et al., 2005). The edible coating prevents fish fillets from changing color and keeps them lighter. Moreover, the colorimetric results of the samples agreed well with the microbial tests (Figure 1), TVB‐N, and TBARS (Table 1) results. Wetterskog and Undeland (2004) reported a direct connection between color changes and the TBARS value during storage time.

### 3.4 Microbial analysis

#### 3.4.1 Mesophilic bacteria growth (TVC)

Most seafood, such as fish, is susceptible to microbial growth. Hence, evaluating the microbial index is an important parameter to determine its quality (Joukar et al., 2023). The microbial growth results of the samples during the storage period in the refrigerator (4°C ± 1) are shown in Figure 1. The first TVC of fish fillets was 3.45 log CFU/g, which showed their freshness (Figure 1a). All samples showed an upward trend in TVC, but the highest growth was shown in the C sample (8.14 log CFU/g), and the least amount was shown in the Ch and AChF1 samples (5.6 log CFU/g) at the end of the period (*p* < .05). The mesophilic growth bacteria in chitosan and LbL‐coated samples were significantly fewer than in C and A samples, which may be due to the antimicrobial features of chitosan. Maghami et al. (2019) reported a decrease in mesophilic bacteria growth in fish fillets coated with chitosan containing fennel essential oil during storage, which was attributed to the synergistic impact of fennel essential oil with chitosan. Mohebi and Shahbazi (2017) reported similar results. The chitosan antimicrobial features are due to the interaction of its cationic agents (NH_3_^+^) with the negative charge of the cell membrane of microorganisms (Valizadeh et al., 2019).

#### 3.4.2 Psychrophilic bacteria growth (TPC)

In fact, TPC (especially aerobic psychrophilic gram‐negative) is the most important group of microorganisms responsible for fish spoilage during cold storage (Sallam, 2007). As shown in Figure 1b, the first TVC of samples was 3.18 log CFU/g, corresponding to the normal range of TPC in fresh fish fillets (2–6 log CFU/g) in other studies (Chytiri et al., 2004). The samples that were coated showed lower TPC, and the C and AChF1 samples had the least amount and most amount of TPC (9.38 and 6.94 log CFU/g, respectively) at the end of the storage time (*p* < .05). Coatings can be a barrier against oxygen transfer, hindering bacterial growth. These results behaved in the same way in the research of Chamanara et al. (2013) on *Oncorhynchus mykiss* fish fillet coated with chitosan and Ebadi et al. (2019) on *Nemipterus japonicus* fish fillet, which is coated with chitosan nanoparticles and also beeswax extract.

Seaweed has some compounds, such as proteins, phenols, sulfated polysaccharides and sulfur functional groups, that are able to control bacterial growth by stopping cell division (Alagappan et al., 2010). Palanisamy et al. (2019) have attributed the structure of the antibacterial effect of fucoidan extracted from *Sargassum polycystome* to the existence of glycoprotein receptors in polysaccharides that react with the mixtures in the cell wall, membrane of the cytoplasm, and DNA of bacteria and cause disturbance in the permeability of the cytoplasmic membrane and, as a result, protein leakage and damage to DNA.

#### 3.4.3 Lactic acid bacteria (LAB)

Lactic acid bacteria are the facultative anaerobic bacteria, which are the most important spoilage bacteria under anaerobic conditions in the refrigerator (Gómez‐Estaca et al., 2009). The results related to the growth of LAB are shown in Figure 1c. The result showed LAB growth increased during storage. In C and samples, the LAB growth increased from 1.26 log CFU/g on the first day to 5.1 and 5.4 log CFU/g on the last day (*p* < .05). In other treatments, especially in LbL‐coated samples, LAB growth was lower at the final step of storage. The performance of different coatings in inhibiting the growth of LAB was similar to the inhibitory effect of these coatings on TPC and TVC bacteria. Similar results were previously reported by Mohebi and Shahbazi (2017) and Ayrapetyan et al. (2021). However, the results of bacterial growth during storage showed chitosan coating was better than alginate coating, and the coating containing fucoidan showed lower bacterial growth compared to other treatments.

### 3.5 Sensory evaluation

**FIGURE 2:**
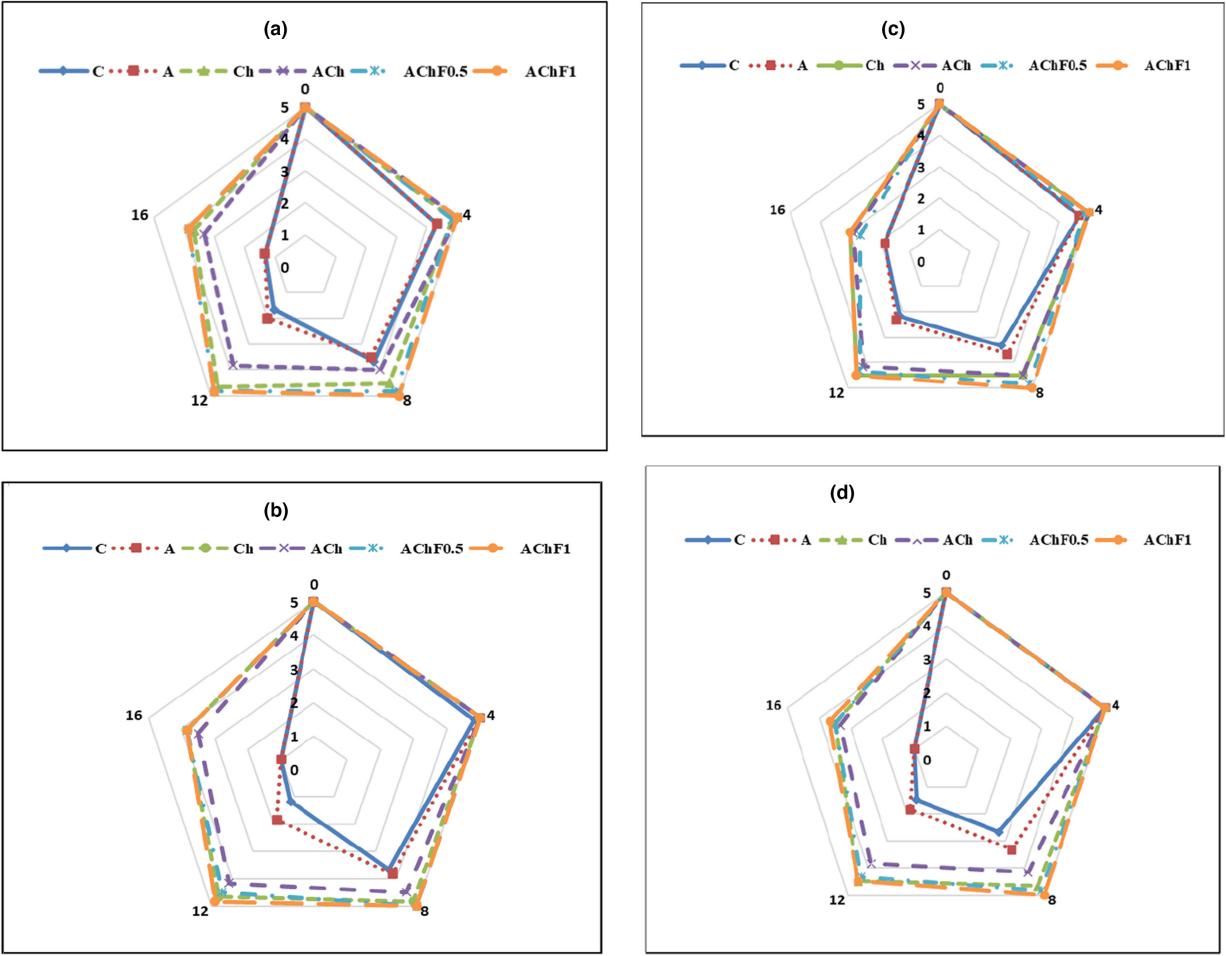
Sensory evaluation of color (a), odor (b), texture (c), and overall acceptability (d) of fish samples during refrigerated storage. C: uncoated fish fillet (negative control), A: fish fillet coated with alginate, Ch: fish fillet coated with chitosan, ACh: fish fillet coated with alginate‐chitosan LbL coating, AChF0.5: fish fillet coated with alginate/fucoidan (0.5%)‐chitosan LbL coating, AChF1: fish fillet coated with alginate/fucoidan (1%)‐chitosan LbL coating. (*n* = 6, Mean ± *SD*).

The sensory evaluation of samples in this research was evaluated based on four factors like color, odor, texture, and total acceptability (Figure 2). Based on the semi‐trained panelists’ observations, the C and A samples had the highest color changes, while the AChF0.5 and AChF1 samples had the lowest changes (Figure 2a). By increasing the storage period of the samples, almost all of them maintained their color status until the fourth day, but after that, there was a significant difference among samples in color characteristics. During the storage period, the most unpleasant odor and texture changes in the C and A samples were observed, while other samples had less odor and texture changes significantly (Figure 2b,c). The highest overall acceptability was attributed to the AChF0.5 and AChF1 samples, and the lowest to the C one (Figure 2d). This is probably because of the function of sulfated groups of fucoidan in preventing oxidation and the antimicrobial properties of chitosan, which can prevent tissue destruction, color, and odor changes. The sensory evaluation results of the samples were in accordance with their chemical and microbial results (Table 1 and Figure 1). The obtained results of this study were similar to Kulig et al. (2017) reports, which were about studying the effect of chitosan‐alginate film on cooked pork.

## 4 CONCLUSION

In conclusion, the findings of the study demonstrate that the use of chitosan‐alginate LbL coatings containing sulfated polysaccharides can effectively enhance the shelf life of rainbow trout fillets during refrigerated storage. Fish fillets coated with AChF1 had a lowered rise in microbial growth (TVC, TPC, and LAB), pH value, TBARS, and TVB‐N content, and physical changes within 16 days. The fish fillets coated with AChF1 showed acceptable sensory characteristics until the 12th day of refrigerated storage. Therefore, the application of the LbL method with chitosan and fucoidan coatings presents a hopeful approach for extending the shelf life of fish fillets and other seafood products under refrigerated conditions.

## AUTHOR CONTRIBUTIONS

**Fatemeh Khorami:** Funding acquisition (equal); investigation (equal); methodology (equal); writing – review and editing (equal). **Sedigheh Babaei:** Conceptualization (equal); investigation (equal); methodology (equal); resources (equal); software (equal); supervision (equal); writing – original draft (equal); writing – review and editing (equal). **Shahriyar Valizadeh:** Validation (equal); writing – original draft (equal). **Mahmood Naseri:** Data curation (equal); investigation (equal); methodology (equal); project administration (equal); resources (equal); software (equal). **Mohammad‐Taghi Golmakani:** Conceptualization (equal); data curation (equal); formal analysis (equal); methodology (equal); project administration (equal); writing – review and editing (equal).

## CONFLICT OF INTEREST STATEMENT

The authors declare that they have no known competing financial interests or personal relationships that could have appeared to influence the work reported in this paper.

## Data Availability

Data sharing is not applicable to this article as no new data were created or analyzed in this study.
